# Shock Wave-Induced Degradation of Polyethylene and Polystyrene: A Reactive Molecular Dynamics Study on Nanoplastic Transformation in Aqueous Environments

**DOI:** 10.3390/molecules30102164

**Published:** 2025-05-14

**Authors:** Tomasz Panczyk, Marcin Cichy, Monika Panczyk

**Affiliations:** 1 Jerzy Haber Institute of Catalysis and Surface Chemistry, Polish Academy of Sciences, ul. Niezapominajek 8, 30239 Cracow, Poland; 2 Department of Chemical Technology, Institute of Chemical Sciences, Faculty of Chemistry, Maria Curie-Sklodowska University in Lublin, pl. Maria Curie-Sklodowska 3, 20031 Lublin, Poland; marcin.cichy@umcs.pl (M.C.); monika.panczyk@umcs.pl (M.P.)

**Keywords:** polyethylene, polystyrene, degradation, nanoplastic, shock, ReaxFF

## Abstract

Degradation of polyethylene and polystyrene was studied theoretically using reactive molecular dynamics based on the ReaxFF force field. The degradation reactions were carried out on nanoparticles (approximately 2 nm in diameter) composed of ideal low-density polyethylene and polystyrene in the presence of water. The reactions leading to degradation were triggered by applying a shock wave through the simulation box. This approach allowed the energy to be transferred to the sample in a controllable manner and initiated the reactions. The state of the nanoparticles after the shock wave passage was investigated in detail, focusing on the type and quantities of new surface functional groups and new chemical connections in the bulk samples. It was found that polyethylene predominantly reveals surface hydroxyl groups (some of which can be protonated) and has the ability to release linear polyhydroxy alcohols. Other surface functional groups with significant presence are ether groups. The degradation of polystyrene proceeds through the addition of hydroxyl groups primarily to the benzene rings, causing their dearomatization. The number of hydroxyl groups in a single ring increases with the degree of degradation, and some hydroxyl groups are also protonated. Polystyrene is also susceptible to crosslink formation, mainly between aromatic rings, leading to branched and dearomatized forms that are chemically distinct from styrene.

## 1. Introduction

Analysis of plastic degradation mechanisms and their products has been a research focus since the 1960s, with explosive growth since 1991, particularly on enzyme-mediated biodegradation by microorganisms and insects [1]. The physicochemical processes through which plastic materials degrade often lead to potentially harmful byproducts, highlighting the urgent need for efficient degradation strategies [2,3].

Polyethylene, one of the most resistant plastics to biodegradation, accumulates in the environment, posing significant threats and contributing to pollution. The slow degradation kinetics of current biotic or abiotic processes and the lack of complete mineralization present challenges in mitigating polyethylene’s environmental impact [4]. Ultraviolet (UV) radiation, heat, and mechanical forces can accelerate the oxidative degradation of polyethylene molecular bonds, causing depolymerization and further degradation. Various methods have been employed to study polyethylene degradation, including thermogravimetric analysis (TGA), Fourier-transform infrared spectroscopy (FTIR) to analyze degraded polymer samples, and Raman spectroscopic measurements to characterize the initial stages of polyolefin degradation [5,6,7].

The degradation of polystyrene involves complex mechanisms influenced by environmental factors such as UV radiation and temperature. Thermal degradation of polystyrene mainly involves homolytic reactions of carbon–carbon bonds, hydrogen transfer reactions, and free radical termination reactions, leading to the formation of products such as styrene, toluene, and ethylbenzene. Photodegradation of polystyrene can result in the generation of water-soluble degradation products, which may contribute to increased toxicity upon degradation [8,9]. Studies have utilized various methods to investigate polystyrene degradation, including density functional theory, X-ray photoelectron spectroscopy, Fourier-transform infrared spectroscopy, and gel permeation chromatography [9,10].

The degradation and breakdown processes of polymers are notably complex in nature, posing challenges for both experimental and computational understanding. Consequently, computational methods have been applied for this purpose infrequently. A combination of density functional theory (DFT) calculations and classical molecular dynamics simulations was used to propose atomistic mechanisms for the formation of common chemical defects in polyethylene [11]. Reactive molecular dynamics simulations were also employed to extract molecular evolution and macroscopic state variables as a function of temperature, temperature ramp rate, and density, using the ReaxFF potential [12]. DFT methods have been used to study the reaction mechanism of thermal degradation of polystyrene. Kinetic parameters such as activation energy and thermodynamic parameters of each thermal degradation pathway have been obtained through theoretical calculations, providing insights into the major formation mechanisms of degradation products [10]. A detailed mechanistic model was also developed and implemented using a kinetic Monte Carlo approach to describe the kinetic degradation dynamics of polystyrene through pyrolytic processes [13].

In our recent publications, we utilized reactive molecular dynamics studies based on the ReaxFF potential. In ReaxFF, the progression of a chemical reaction—whether it proceeds forward or reverses—is not explicitly guided by a predefined reaction coordinate. Instead, it emerges from the local chemical environment, including bond orders, interatomic distances, and angles evaluated at each timestep and averaged in some time window [14,15] to analyze degradation reactions occurring in low-density polyethylene (LDPE) [16], polyethylene terephthalate (PET) [17], polypropylene (PP), and polystyrene (PS) [18], in a water-free environment. By mechanically cleaving bulk samples, we identified and classified surface functional groups that can develop on the surfaces of plastic materials. Additionally, we employed shock compression as a means of activating chemical reactions that would typically occur over prolonged timescales in the natural degradation of polymers. Molecular dynamics simulations have been used to explore shock-induced chemical reactivity, indicating extensive and complex chemical reactions at elevated pressures and temperatures [19]. Reactive molecular dynamics simulations have also demonstrated that intramolecular strain energy induced by shock waves accelerates chemical kinetics, providing evidence that shock compression affects the activation energy of reactions [20].

The current study focuses on analyzing the degradation mechanisms of LDPE and PS nanoparticles in a water environment, which is a more common scenario in a natural environment. From a computational perspective, the presence of water introduces additional complexity, as the reactions become more intricate. However, the resulting surfaces of the nanoparticles will be more representative of naturally formed nanoplastics. The computational methodology in this study examines the likelihood of reactions with gradually increasing activation energies, providing insights into the composition of degraded materials subjected to various levels of external energy absorption. Although shock compression is not a natural environmental factor leading to plastic degradation, from a computational standpoint, it induces effects similar to those of UV radiation or mechanical stress by activating and accelerating reactions that would not occur without such activation in the given chemical environment.

In this study, we investigated the degradation behavior of two widely used plastics—LDPE and PS—chosen for their structural simplicity and prevalence as plastic waste. Our molecular simulations revealed that PS begins to degrade under milder conditions compared to LDPE, indicating that PS may degrade more rapidly and easily under environmental conditions as well. A key outcome of our work is the detailed characterization of surface transformations in both materials. We observed the formation of various oxygen-containing functional groups, with their types and populations analyzed comprehensively. Additionally, our results suggest that during degradation, plastic particles release small organic molecules and develop internal crosslinks. To our knowledge, no experimental studies have so far provided such detailed insight into the possible functional groups and their distributions on the surfaces of degrading LDPE and PS. Thus, the findings presented in this publication offer a unique and valuable in situ perspective—based on molecular-level simulations—on the chemical evolution of these materials under degradation.

The manuscript is organized as follows. Section 2, divided into relevant subsections, describes both materials separately, given their differing chemical structures and degradation reactions. The description of the generation of ideal LDPE and PS nanoparticles precedes the analysis of surface compositions, making the discussion clearer. In Section 4, we compare the most essential findings for both materials and highlight the differences in their chemical compositions as predicted by our computational study.

## 2. Results and Discussion

### 2.1. Preparation of the LDPE Nanoparticle

The description of the preparation of an ideal LDPE nanoparticle is as follows. Briefly, we started with an initially linear but branched chain of -CH_2_- groups [16]. The chain consisted of 975 carbon atoms, with six of them being branched, which is representative of the molecular structure of LDPE [21]. The total number of atoms was 2927, with a molecular mass of 13,690 g/mol, slightly below the lower limit of the number average molecular weight value for LDPE [22]. The obtained atomic structure was then subjected to preliminary molecular dynamics (MD) to fold it into a compact, roughly spherical nanoparticle. The folding of the LDPE chain was carried out at a temperature of 300 K under NVT ensemble conditions in vacuum. As a result, the polymer spontaneously adopted a random structure representative of an amorphous polymer nanoparticle. This stage of MD calculations was performed using the standard Amber force field, and the parameter values were generated using the AcPyPE script [23].

The atomic coordinates of the obtained ideal LDPE nanoparticle were utilized in constructing the simulation box for conducting chemical reactions. Initially, the nanoparticle underwent equilibration with the ReaxFF force field, excluding water, at a temperature of 300 K. The force field parameter set was obtained from reference [24] (CHON-2017_weak), where the parameters from the water branch were improved to better describe water/organic mixtures. The ReaxFF parameters were neither modified nor re-validated in this study, as the parameter set applied had already undergone validation. It originates from the earlier protein–water parameter set (protein-2013) [25], which was subsequently refined to enhance the description of hexane–water mixtures, ethanol solvation, and the degradation of tetramethylammonium (TMA) in liquid water.

**Figure 1:**
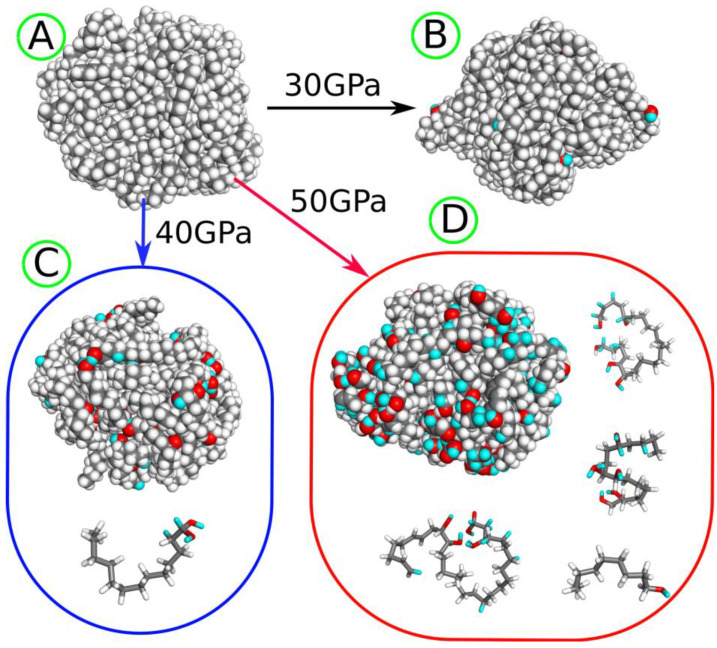
(**A**) LDPE nanoparticle before degradation and (**B**) after shock compression to 30 GPa. (**C**) The LDPE nanoparticle and fragmentation product tridecane-1,1-diol generated after shock compression to 40 GPa. (**D**) The LDPE nanoparticle and several fragmentation products obtained after shock compression to 50 GPa. The gray spheres and sticks represent carbon atoms, while white spheres and sticks represent hydrogen atoms, both originating from the LDPE nanoparticle. The red spheres and sticks denote oxygen atoms transferred from water molecules to the nanoparticle as a result of degradation induced by shock compression. The cyan spheres and sticks represent hydrogen atoms transferred from water molecules after shock compression.

Subsequently, the new atomic coordinates of the LDPE nanoparticle were employed for equilibration with bulk water. The initial atomic coordinates of water molecules were generated based on the geometry of the standard TIP3P water model and arranged on a regular lattice within the simulation box. However, during the simulations, the water molecules were no longer treated as TIP3P; they were fully flexible and capable of participating in chemical reactions. A total of 1662 water molecules were inserted, and the initial box size was set to 55 Å × 55 Å × 55 Å. This configuration is evidently far from thermodynamically relaxed, and to prevent undesired reactions at this stage, the reactivity of the LDPE nanoparticle was suppressed by imposing constraints, specifically applying rigid body motion constraints for the LDPE nanoparticle. After completing the run in the NPT ensemble (12.5 ps), the water phase was allowed to relax and brought to a temperature of 300 K. The resulting configuration shown in Figure 1A, relaxed and reorganized under the ReaxFF potential, has effectively erased any structural memory of the prior force field (Amber). Thus, that LDPE nanoparticle, surrounded by the water phase, was ready to run reactive dynamics after conducting a short re-equilibration run without constraints. A verification of the polymer density, prepared in a similar manner, was conducted in our previous study, showing good agreement with experimental data [16].

### 2.2. Conducting Degradation Reactions of LDPE Nanoparticle in Water

At normal pressure and temperature conditions, such as 300 K and 1 bar, LDPE, like other plastics, typically does not react with water. However, plastic degradation can occur due to prolonged exposure of the material to light and moisture. UV radiation can induce the formation of radicals, which in turn initiate chain scissions, crosslinking, or oxidative degradation, especially in the presence of an oxygen source [26,27,28]. From a fundamental perspective, UV radiation delivers external energy, facilitating these reactions. As already mentioned, this energy input in molecular dynamics simulations can be provided by the shock compression of the sample [16,17]. Therefore, controlled compression of molecules, when described by a reactive force field, may induce reactions in areas most susceptible to chemical transformations.

Thus, the LDPE nanoparticle, surrounded by reactive water as described in the previous section, has been subjected to shock compression by running simulations with a specialized barostat, known as a hugoniostat [29,30], implemented in LAMMPS [31]. The hugoniostat dynamics simulate the passage of a shock wave through the sample, and depending on the settings, the system is compressed to the target pressure and instantaneously heated to high temperature. These calculations were performed for several target pressures to determine the conditions under which the reaction with water begins and to gradually increase the degree of oxygen-containing groups formed on the surface of the LDPE nanoparticle.

Performing shock compression with target pressures up to 30 GPa did not induce any chemical rearrangements of atoms, but only physical compression of the sample. The first reactions begin at a compression pressure of 30 GPa. Figure 1 provides visual representations of the LDPE nanoparticle before (Figure 1A) and after shock compression (Figure 1B–D). To distinguish between hydrogen atoms originating from water and those present initially, the cyan color is used for hydrogen atoms transferred from water molecules.

At 30 GPa, the nanoparticle preserves its integrity and develops a small number of hydroxyl groups on the surface. Higher compression pressures lead to the fragmentation of the original nanoparticle; at 40 GPa, a small molecule (tridecane-1,1-diol) detaches from the nanoparticle, while at 50 GPa, many light molecules are generated, with the four heaviest illustrated in Figure 1D. Still higher compression pressures result in the very strong destruction of the nanoparticle; thus, they are not further considered.

As observed in Figure 1, increasing the compression pressure leads to the development of more oxygen-containing surface groups and medium to small molecule alcohols with multiple hydroxyl groups, revealing unsaturated bonds as well. These alcohols can be water-soluble and lead to widespread contamination of water, aside from the presence of the more hydrophilic plastic nanoparticle itself.

Table 1 summarizes the most essential findings representative of the growing degradation levels of the LDPE nanoparticle. The primary conclusion drawn from the analysis of Table 1 is that the number of new functional groups generated during degradation is relatively small. Specifically, six types of oxygen-containing groups have emerged, along with a new connection formed by the simple detachment of hydrogen atoms leading to double bonds, C3.

**Figure 2:**
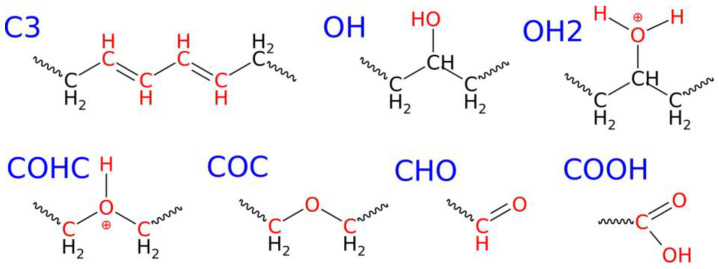
Structural formulas of the new chemical connections formed during the degradation of the LDPE nanoparticle under shock compression in water.

These newly formed oxygen-containing groups include hydroxyl (OH), leading to the formation of alcohols; protonated hydroxyl (OH2); ether (COC); protonated ether (COHC); aldehyde (CHO); and carboxyl (COOH). The populations of these groups vary significantly and depend on the degradation level/compression pressure. Therefore, the most prevalent group appearing during LDPE degradation in the presence of water is likely hydroxyl groups. Their detailed placement within the molecule is illustrated in Figure 2, where it can be observed that these groups lead to the formation of secondary alcohols. The occurrence of hydroxyl groups at primary positions was only incidental. This indicates that water molecules are capable of substituting hydrogen atoms with hydroxyl groups in the polymer chain. Additionally, this process is most feasible as the formation of hydroxyl groups initiates first under the mildest conditions, at 30 GPa. However, detailed reaction pathways leading to the formation of these functional groups, including their protonated forms and the specific mechanisms involved, cannot be extracted from ReaxFF simulations. Such insights would require full quantum chemical calculations on simplified model systems.

The second most active process involves the generation of C3 connections. These occur when a carbon atom loses one hydrogen, resulting in the emergence of a double bond (or radical). These types of connections can occur in cascades, forming a sequence of double bonds, as illustrated in Figure 2. Each carbon atom with a double bond is counted individually, so the scenario depicted in Figure 2 corresponds to four individual C3 connections, which are summed and presented in Table 1. Situations where one carbon atom loses two hydrogen atoms or attaches an extra hydrogen were not observed.

Double bonds and/or radicals also appear during the degradation of plastics in the absence of water [17,18]. Therefore, the formation of C3 connections results here from the activation of CH_2_-CH_2_ bonds, leading to the removal of hydrogen atoms due to energetic excitation. The released hydrogens in a wet environment then participate in subsequent reactions with water molecules, forming ions.

The third most abundant functional group formed is either ether or protonated hydroxyl, depending on the compression pressure. However, both of these groups are definitely less abundant compared to the OH or C3 populations. Nevertheless, it is worth noting that protonated hydroxyls can appear on the nanoparticle surface during its degradation in a wet environment, and the presence of ether groups is expected as well.

The formation of ether groups is particularly interesting because it involves the splitting of alkane chains and the subsequent insertion of oxygen from water molecules. The population of ether groups is significant only at the highest compression pressure studied, indicating that this reaction requires a very strong activation of carbon–carbon bonds and the presence of a water molecule. A similar mechanism is expected for the formation of aldehyde and carboxyl groups. Both require the cleavage of a C–C bond in the alkane chain and the substitution of oxygen and hydrogen atoms from water molecules. Therefore, their populations are small and observed only at the highest compression pressure of 50 GPa.

The physical properties of the nanoparticle are also affected by shock compression. Factors such as gyration radius, solvent-accessible surface area, and nanoparticle mass change as the compression pressure increases. The gyration radius serves as a measure of nanoparticle size, although for nonspherical objects, it is typically smaller than their spatial size. As observed, the R_g_ changes only slightly, but it definitely increases with compression pressure up to 40 GPa. Subsequently, it decreases to the smallest value due to significant fragmentation. Interestingly, the addition of oxygen atoms leads to an increase in nanoparticle mass and size, even in the case of a 40 GPa compression pressure where a relatively large molecule was detached from the nanoparticle. A similar trend is observed for solvent-accessible surface area, which represents the external surface area of the nanoparticle. It increases with degradation due to a larger number of attached oxygen atoms; however, it decreases after significant fragmentation occurring at a compression pressure of 50 GPa.

**Figure 3:**
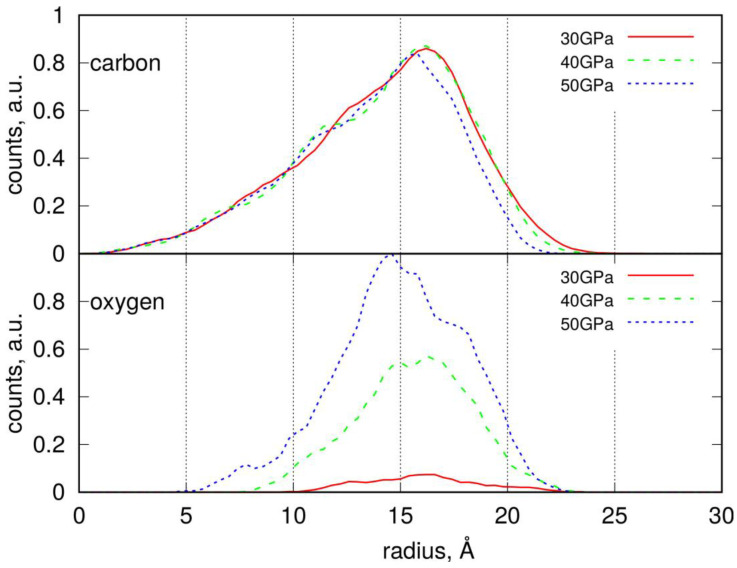
Radial distribution of carbon and oxygen atoms within the degraded LDPE nanoparticles determined by counting the number of atoms in a spherical slice of thickness Δr at a distance r from the center of mass.

The real size of the nanoparticles can be deduced from the analysis of Figure 3. The top part of this figure displays the occurrence of carbon atoms in spherical slices with a thickness of 0.4 Å at varying distances, r, from the nanoparticle’s center of mass. This distribution reveals a maximum, indicating the distance at which the number of atoms begins to decrease. For nonspherical objects, this distance indicates when a probe sphere is no longer completely filled with atoms, and void space begins to increase. Thus, a good measure of the true size is the distance where the steepest descent occurs. So, the nanoparticles compressed at 30 and 40 GPa have radii of approximately 18 Å, while those compressed at 50 GPa are slightly smaller, around 17 Å. An analogous distribution of oxygen atoms allows us to determine the localization of functional groups. As observed, the positions of maxima for carbon and oxygen atoms nearly coincide, indicating that functional groups are located on the external surface of the nanoparticle; no oxygen-containing groups are present in the central part of the nanoparticle. The localization of oxygen atoms for nanoparticles compressed at 50 GPa reveals a significant number of counts at distances of 5–10 Å; however, these counts appear as a result of fluctuations in nanoparticle shape. The surface density of the functional groups can thus be estimated by dividing their populations by the SASA value.

An important observation is the type of small- and medium-sized molecules detached from the LDPE nanoparticle during degradation. This phenomenon occurs under a compression pressure of 40 GPa, i.e., at significant material degradation. Stronger degradation, occurring at 50 GPa pressure, results in the severe fragmentation of the nanoparticle and the formation of numerous different products. Analysis of the structural formulas of these molecules leads to the conclusion that they are mainly secondary alcohols with multiple hydroxyl groups. Many of them also feature hydroxyl groups at primary positions, indicating molecular structures typical of surfactants. Additionally, ethyl alcohol and methane were observed as possible degradation products of LDPE under wet conditions. The presence of molecules with multiple polar hydroxyl groups poses additional risks as they are water-soluble and may be widely distributed in aquatic systems.

### 2.3. Preparation of PS Nanoparticle

The pristine polystyrene nanoparticle was prepared according to the following procedure. The monomer structure was manually created and subjected to polymerization using the tleap program from the AmberTools package [32]. In this case, the number of monomer segments was 183, giving the molecular mass of the molecule as 20,130 g mol^−1^. The initial state structure of the PS molecule was a linear chain, with all C_6_H_5_ groups oriented on the same side of the polymer backbone. This chain underwent an initial dynamics simulation to fold it into a nanoparticle with roughly spherical symmetry. The folding of the PS chain was carried out at a temperature of 300 K under NVT ensemble conditions in vacuum. As a result, the polymer spontaneously adopted a random structure representative of an amorphous polymer nanoparticle. This stage of MD calculations was performed using the standard Amber force field, and the parameter values were generated using the AcPyPE script [23].

In the next step, the nanoparticle underwent equilibration with the ReaxFF force field, excluding water, at a temperature of 300 K. The force field parameter set was obtained from reference [24] (CHON-2017_weak). The second equilibration step involved water: the equilibrated PS nanoparticle was placed in the center of a cubic box, and the remaining space was filled with 3459 water molecules. The NPT run at 300 K and 1 bar finally resulted in a configuration with the relaxed PS nanoparticle surrounded by a water phase and in an intact chemical state. This resulting configuration, relaxed and reorganized under the ReaxFF potential, has effectively erased any structural memory of the prior force field (Amber). This configuration was used as the starting point for further reactive dynamics runs with degradation reactions. A verification of the polymer density, prepared in a similar manner, was conducted in our previous study, showing excellent agreement with experimental data [18].

### 2.4. Conducting Degradation Reactions of PS Nanoparticle in Water

Similarly to the case of LDPE described in Section 2.2, the PS nanoparticle was subjected to shock compression to initiate reactions with water and within the polymer bulk phase. The compression pressure was controlled by the hugoniostat (nphug barostat implemented in LAMMPS [31]) and was set to 10, 20, 30, and 40 GPa. It was found that compressions around 10 GPa did not lead to any chemical transformations. The first reactions started at 20 GPa and involved surface atoms and water molecules. Thus, further analysis concerned compressions of 30 and 40 GPa, which led to increasingly intense chemical transformations at the surface and within the bulk of the PS nanoparticle.

**Figure 4:**
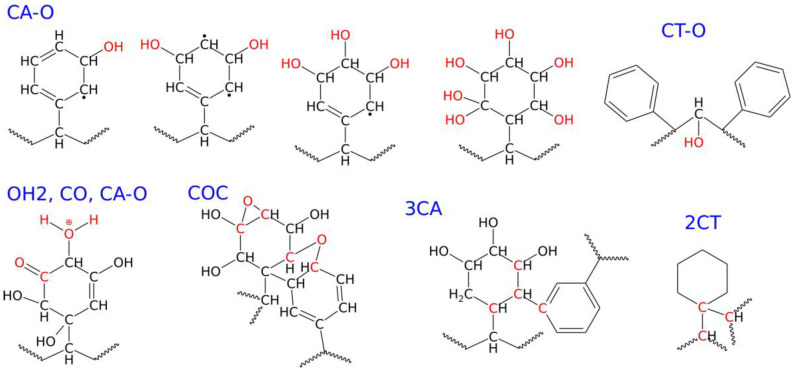
Structural formulas and definitions of the new chemical connections formed during the degradation of the PS nanoparticle under shock compression in water.

Table 2 shows the essential results obtained during the degradation of PS nanoparticle in water, simulated by running shock wave passages at various compression pressures. The analysis of Table 2 should be accompanied by a simultaneous look at Figure 4, as that figure helps to understand which chemical structures are associated with the new chemical connections presented in Table 2.

**Figure 5:**
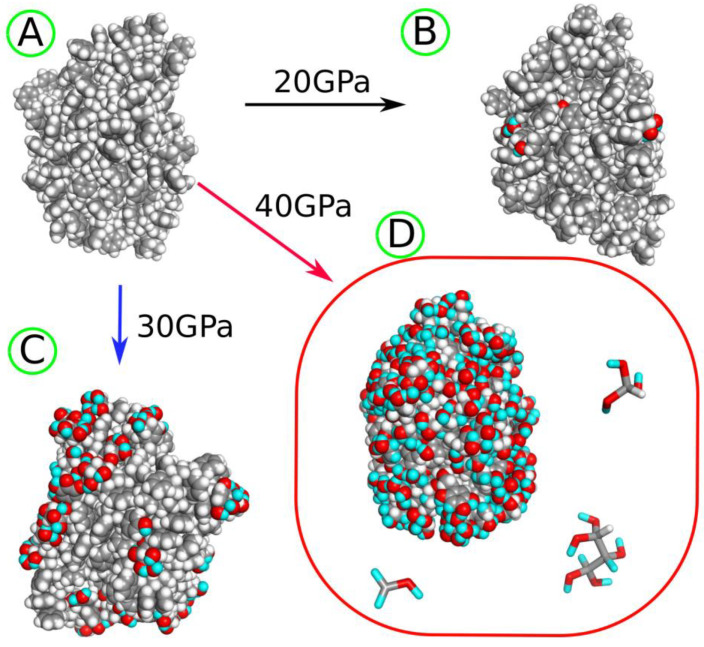
(**A**) PS nanoparticle before degradation and (**B**) after shock compression to 20 GPa. (**C**) The PS nanoparticle after shock compression to 30 GPa. (**D**) The PS nanoparticle and several fragmentation products obtained after shock compression to 40 GPa. The gray spheres and sticks represent carbon atoms, while white spheres and sticks represent hydrogen atoms, both originating from the PS polymer. The red spheres and sticks denote oxygen atoms transferred from water molecules to the nanoparticle as a result of degradation induced by shock compression. The cyan spheres and sticks represent hydrogen atoms transferred from water molecules after shock compression.

As seen in both Table 2 and Figure 4, the most frequent chemical event is the addition of oxygen, coming from water, to the polymer molecule. It is interesting to note that the addition of oxygen occurs mainly at the aromatic ring, and this type of new chemical connection is labeled as CA–O (CA—aromatic carbon). It can also happen at the polymer backbone, CT–O (CT—tertiary carbon), but the occurrence of such an event is much lower than CA–O, as seen in Table 2. CA–O connections mainly involve the addition of hydroxyl groups, leading to species with structural formulas shown in Figure 4. These formulas are rather symbolic, but they increasingly appeared with the rising compression pressure. The number of hydroxyl groups linked to a single ring varies with the compression pressure, but the first three compounds, with one to three -OH groups, appeared already at 20 GPa. The highly hydroxylated (fourth in Figure 4) compound was found at 30 GPa. As seen in Figure 4, these hydroxylated rings are often left with unpaired electrons and are thus radicals. Thus, the first important conclusion is that the PS nanoparticle, upon degradation in the presence of water, attaches hydroxyl groups to its surface, and the density of these groups depends on the degradation level. Figure 5 shows how densely the hydroxyl (or other oxygen-containing groups) are distributed on the surface of the PS nanoparticle, depending on the degradation level.

The other oxygen-containing groups found on the surface of the degraded PS nanoparticle are listed in Table 2. These include protonated hydroxyls (OH2), epoxy or ether (COC), carbonyl (CO), aldehyde (CHO), and carboxylic acid (COOH). They appeared at higher compression pressures with varying populations: the most frequent are protonated hydroxyls, COC, and CO. The other groups, particularly aldehyde and carboxylic acids, are rare. It is also worth mentioning that these oxygen-containing groups are exclusively localized on the surface; no such groups were found in the bulk of the nanoparticle.

Another important structural factor resulting from degradation is the appearance of crosslinks. These can be of a very complex nature [17,18], but here we will focus on an easily definable factor: the number of connections formed by an aromatic carbon with either three other aromatic carbons (3CA) or two tertiary carbons (2CT), as shown in Figure 4. Analysis of the populations of these two types of crosslinks allows us to conclude that the formation of crosslinks between aromatic rings is more facile than with tertiary carbons.

The PS nanoparticle, as seen in Figure 5A, took on an ellipsoidal shape at the very beginning during equilibration runs without shock compression. That shape was actually preserved at all stages of the degradation, but the surface of the nanoparticle was gradually modified by oxygen-containing species. This led to significant changes in the nanoparticle size, which is roughly described by the values of gyration radii in Table 2. Compression to 20–30 GPa led to some increase in nanoparticle size, mainly due to the addition of OH groups. However, after compression to 40 GPa, the nanoparticle size significantly decreased. This effect resulted from the development of crosslinks, 3CA and 2CT, which led to the contraction of the nanoparticle and the stiffening of its structure. A similar trend is seen in the SASA values: a small increase at moderate compressions and a significant decrease at 40 GPa, resulting from the development of crosslinks during strong compression. Thus, the highest compression pressure applied is probably an overkill in modeling degradation processes induced by external energy transfer, such as UV radiation.

The molecular masses and compositions of the PS nanoparticles show that the degradation of PS in water leads to the addition of oxygen and hydrogen atoms originating from water. Only at the highest compression pressure do we observe the detachment of small molecules containing polymer fragments, but the effective mass of the nanoparticle is still larger overall. The fragmentation of PS leads to the formation of very light products, which are linear polyhydroxy alcohols. However, the fragmentation of PS is not facile, as it started at compression pressures of 40 GPa, which are probably not representative of the natural degradation of PS in water.

**Figure 6:**
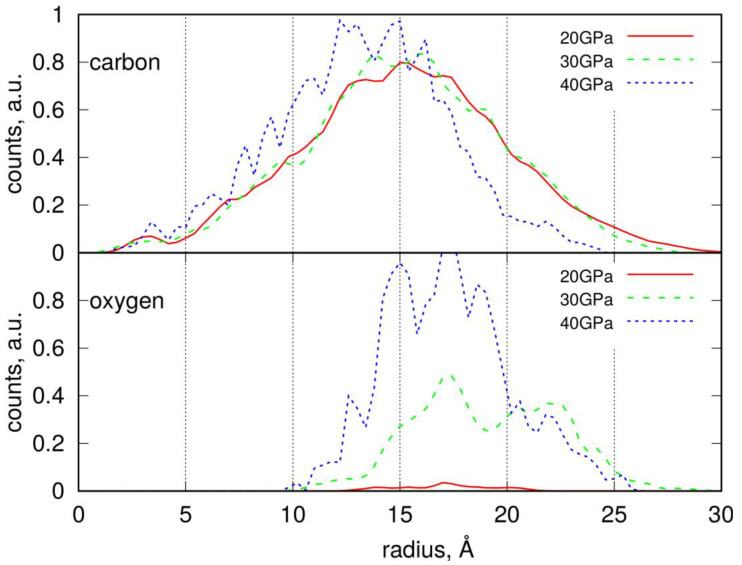
Radial distribution of carbon and oxygen atoms within the degraded PS nanoparticles determined by counting the number of atoms in a spherical slice of thickness Δr at a distance r from the center of mass.

Radial distribution functions (RDFs) for PS nanoparticles, determined analogously to those for LDPE NPs, are presented in Figure 6. These distributions exhibit a clear maximum, indicating the distance at which the atomic density begins to decrease. Given that the PS nanoparticles are highly non-spherical—as clearly shown in Figure 5—this characteristic distance marks the point where a probe sphere is no longer fully occupied by atoms and void regions begin to appear. Therefore, the most appropriate measure of the nanoparticle’s effective size is the point at which the steepest decline in the RDF occurs, similarly to the LDPE case.

However, due to the pronounced non-spherical shape of PS nanoparticles, the RDFs exhibit a form of oscillation. This is a result of distance averaging during the free rotational motion of the nanoparticles around their centers of mass.

The average radius of PS nanoparticles is approximately 20 Å, with those subjected to compression at 40 GPa being slightly smaller, around 17–18 Å. The distribution of oxygen atoms allows us to identify the localization of functional groups. As shown, the oxygen atoms are concentrated at the outer surface of the nanoparticles, indicating that functional groups are predominantly located there. Moreover, the density of these functional groups significantly increases with compression pressure. The surface density of the functional groups can be estimated by dividing their respective counts by the solvent-accessible surface area (SASA), if needed.

## 3. Methods

The simulations were performed using the Large-scale Atomic/Molecular Massively Parallel Simulator (LAMMPS, 29 August 2024—Update 1) [31]. Input file preparation, including atomic coordinates, was carried out with custom-designed scripts and the tleap program from AmberTools22 [32] for polymerization, alongside the Resp Esp Charge Derive Server [33] for computing monomer point charges. These tools played a crucial role in setting up the simulation box, arranging polymer molecules, and ensuring proper equilibration. The production runs were executed using the ReaxFF force field, specifically its latest implementation for CHO systems within the water branch [24]. The charge equilibration was handled through the Qeq method [34], with input parameters derived from the ReaxFF potential file. The equations of motion were integrated with a timestep of 0.25 fs.

## 4. Summary and Conclusions

The application of shock compression at various pressures allowed us to mimic the energy transfer that plastic nanoparticles experience in natural environments, primarily from UV radiation or mechanical stress. Prolonged sunlight exposure leads to faster and more intense degradation, corresponding to higher energy levels, which we simulated by applying increased compression pressures. By gradually increasing the compression pressure, we were able to investigate the reactions likely to occur readily. These reactions are discussed at the lowest compression pressures, as even lower pressures did not induce any reactions. The highest compression pressures studied here allowed us to identify chemical structures of highly degraded materials; however, we do not claim that such states are achievable under natural degradation conditions. Nonetheless, by observing chemical reactions occurring under progressively increasing compression pressures, we can draw important conclusions about the types and likelihood of their occurrence, which should align with the data in Table 1 and Table 2.

The most important conclusions that can be drawn from the results of LDPE degradation are the predominant presence of surface hydroxyl groups (some of which can be protonated) and the ability to release linear polyhydroxy alcohols. Other surface functional groups with significant presence are ether groups. The bulk material is modified by the detachment of hydrogens and the generation of unsaturated bonds.

The degradation of PS proceeds through the addition of hydroxyl groups primarily to the benzene rings, causing their dearomatization. The number of hydroxyl groups in a single ring increases with the degree of degradation, and some hydroxyl groups are also protonated. Other surface species, although with smaller populations, include ether, epoxy, or carbonyl groups. PS is also susceptible to crosslink formation, mainly between aromatic rings, leading to branched and dearomatized forms chemically distinct from styrene.

From the analysis of the minimum compression pressures required to initiate reactions (20 GPa for PS and 30 GPa for LDPE), we can draw conclusions about the relative susceptibility of these materials to degradation in water. PS requires less energy to degrade, and thus its nanoparticles are likely to degrade faster than those of LDPE. This remark also concerns the potential impact of surface energy and interfacial properties on the reactivity of nanoparticles in aqueous environments.

The analysis of the simulation results leads to the conclusion that the surfaces of plastic nanoparticles become significantly altered after degradation in the presence of water and contain numerous oxygen-containing groups. These groups are mainly hydroxyl groups, and when comparing both materials, the population of hydroxyl groups is more dominant in the case of PS than in LDPE. This must lead to their hydrophilization and enhanced solubility, or simply the formation of stable colloid suspensions in water. In such a form, they become easily assimilated by aquatic organisms and thus pose a significant threat to both aquatic life and humans.

## Figures and Tables

**Table 1:** Essential parameters of the LDPE nanoparticle before and after degradation induced by shock compression at various pressures. The new chemical connections are labeled according to standard chemical nomenclature and are presented as structural formulas in Figure 2.

		Initial State	Compression Pressure		
			30 GPa	40 GPa	50 GPa
New chemical connections/ functional groups within/on the nanoparticle	C3	0	0	8	47
	OH	0	7	61	96
	OH2	0	0	2	6
	COHC	0	0	0	1
	COC	0	0	1	16
	CHO	0	0	0	4
	COOH	0	0	0	1
Gyration radius, R_g_, Å		14.66	14.74	14.86	14.51
Solvent Accessible Surface Area, SASA, Å^2^		7091	7152	7376	6944
Stoichiometry and molar mass		C_975_H_1952_; 13,678 g mol^−1^	C_975_H_1943_O_8_*H*_7_; 13,804 g mol^−1^	C_962_H_1835_O_68_*H*_74_; 14,566 g mol^−1^	C_882_H_1440_O_125_*H*_245_; 14,292 g mol^−1^
Other fragmentation products		none	none	C_13_H_24_O_2_*H*_2_	C_30_H_43_O_5_*H*_16_; C_24_H_33_O_6_*H*_12_; C_17_H_28_O_5_*H*_9_; C_9_H_19_O*H*; C_6_H_10_O_2_*H*_3_; C_2_H_4_O_2_*H*_2_; C_2_H_5_O*H*; CH*H*_3_

**Table 2:** Essential parameters of the PS nanoparticle before and after degradation induced by shock compression at various pressures. The new chemical connections are labeled according to standard chemical nomenclature and are presented as structural formulas in Figure 4.

		Initial State	Compression Pressure		
			20 GPa	30 GPa	40 GPa
New chemical connections/ functional groups within/on the nanoparticle	CA–O	0	6	179	319
	CT–O	0	1	8	52
	OH	0	7	156	246
	OH2	0	0	11	55
	COHC	0	0	0	1
	COC	0	0	6	23
	CO	0	0	8	19
	CHO	0	0	0	2
	COOH	0	0	0	1
	3CA	0	0	48	338
	2CT	0	0	0	11
Gyration radius, R_g_, Å		15.74	15.78	16.45	14.68
Solvent Accessible Surface Area, SASA, Å^2^		9250	9173	9632	7471
Stoichiometry and molar mass		C_1546_H_1550_; 20,131 g mol^−1^	C_1546_H_1550_O_7_*H_6_*; 20,249 g mol^−1^	C_1546_H_1484_O_181_*H_181_*; 23,143 g mol^−1^	C_1538_H_1195_O_346_*H*_566_; 25,783 g mol^−1^
Other fragmentation products		none	none	none	C_3_HO_5_*H_7_*; CHO_3_*H_3_*; CO*H_3_*; CHO*H*;

## Data Availability

Data are available upon request.
